# Child-Onset Cerebellar Ataxia Caused by Two Compound Heterozygous Variants in ADPRS Gene: A Case Report

**DOI:** 10.3389/fgene.2021.788702

**Published:** 2022-05-19

**Authors:** Jiehui Ma, Qiaoqiao Qian, Shuang Yan, Haoyu Dou, Cheng Li, Dan Sun

**Affiliations:** ^1^ Department of Neurology, Wuhan Children’s Hospital, Tongji Medical College, Huazhong University of Science & Technology, Wuhan, China; ^2^ School of Medicine, Jianghan University, Wuhan, China; ^3^ Aegicare (Shenzhen) Technology Co., Ltd., Wuhan, China; ^4^ Department of EEG (Electroencephalogram), Wuhan Children’s Hospital, Tongji Medical College, Huazhong University of Science & Technology, Wuhan, China

**Keywords:** CONDSIAS, ADPRS, nonsense-mediated mRNA decay, intron retention, compound heterozygous variant

## Abstract

**Background:** Gene variants of ADP-ribosylserine hydrosylase, also known as ADP-ribosylhydrolase-like 2 (*ADPRS* or *ADPRLH2;* OMIM: 610624), can cause stress-induced childhood-onset neurodegeneration with variable ataxia and seizures (CONDSIAS, OMIM: 618170), an ultra-rare neurodegenerative autosomal recessive disorder. *ADPRS* encodes ADP-ribosylhydrolase 3, which removes poly(ADP-ribose) polymers, whose posttranslational addition occurs under stressful conditions.

**Case Presentation:** After a respiratory tract infection, a 30-month-old male patient presented with unsteady gait that rendered walking impossible without external help. Neurological examination revealed acute cerebellar ataxia, electroencephalogram results were abnormal, and brain magnetic resonance imaging revealed slightly widened cerebellar sulci. Laboratory tests showed decreased levels of thyroid-stimulating hormone, and increased levels of plasma lactic acid and serum cardiac enzymes. The cerebrospinal fluid glucose test was positive. Four months after onset, the patient died of sudden convulsions. Using whole exome sequencing, we identified two novel compound heterozygous *ADPRS* variants: NM_017825.3:c.580C>T (p.Gln194Ter) and NM_017825.3:c.803-1G>A. RNA sequencing indicated that the former mutation might cause nonsense-mediated mRNA decay. The c.803-1G>A variant was found to be a splice-site mutation that leads to the transcriptional retention of intron 5. According to the guidelines of the American College of Medical Genetics and Genomics, the two variants were classified as pathogenic.

**Conclusion:** We present the first report of the existence of two compound heterozygous variants of *ADPRS*, which leads to CONDSIAS.

## Introduction

Stress-induced childhood-onset neurodegeneration with variable ataxia and seizures (CONDSIAS, OMIM: 618170) is a disease that is inherited in an autosomal recessive manner. Patients usually develop the disease before the age of 1 or during childhood. The main clinical manifestations of the disease are neurodegeneration, epilepsy, nystagmus, ocular muscle paralysis, strabismus, ptosis, muscular atrophy of the tongue bundle, reduced muscle fiber size, developmental stagnation, ataxia, articulation disorder, tremor, atrophy of the cerebellum and spinal cord, and autism. Some patients also present with deafness. The ADP-ribosylhydrolase-like 2 gene (*ADPRS* or *ADPRLH2*; NM_017825.2; OMIM: 610624) has been identified to be involved in the etiology of CONDSIAS. This gene, located at position 1p35.3-p34.1, comprises six coding exons, and is translated into the ADP-ribosylhydrolase 3 (ARH3) protein (Hoch and Polo, 2019), which is found in the nucleus, cytoplasm, and mitochondria, where it eliminates the excessive accumulation of poly(ADP-ribose) (PAR). ADP-ribosylation is a significant posttranslational modification that is involved in several essential physiological and pathological processes, such as DNA repair, transcription, telomere function, and apoptosis (Wright et al., 1996; Martinez-Zamudio and Ha, 2012). Upon genotoxic stress, oxidative stress, and mitotic stimulation, PAR levels can increase 10- to 500-folds, preventing cell death in stressful conditions (Zhang et al., 2013). The PAR polymer is then rapidly degraded after removal of the stressor. In fact, excessive PAR accumulation triggers a cascade of cell death responses, initiating the process of parthanatos (Mashimo et al., 2013). Therefore, the presence of a functional ARH3 protein is fundamental in the maintenance of cellular homeostasis. Consequently, it is unsurprising that loss-of-function mutations in *ADPRS* can lead to CONDSIAS (Danhauser et al., 2018). Here, we report two novel compound heterozygous *ADPRS* variants (c.580C>T, c.803-1G>A) in a child with CONDSIAS, with the intent to provide additional information for the diagnosis of this disease.

## Case Description

**FIGURE 1:**
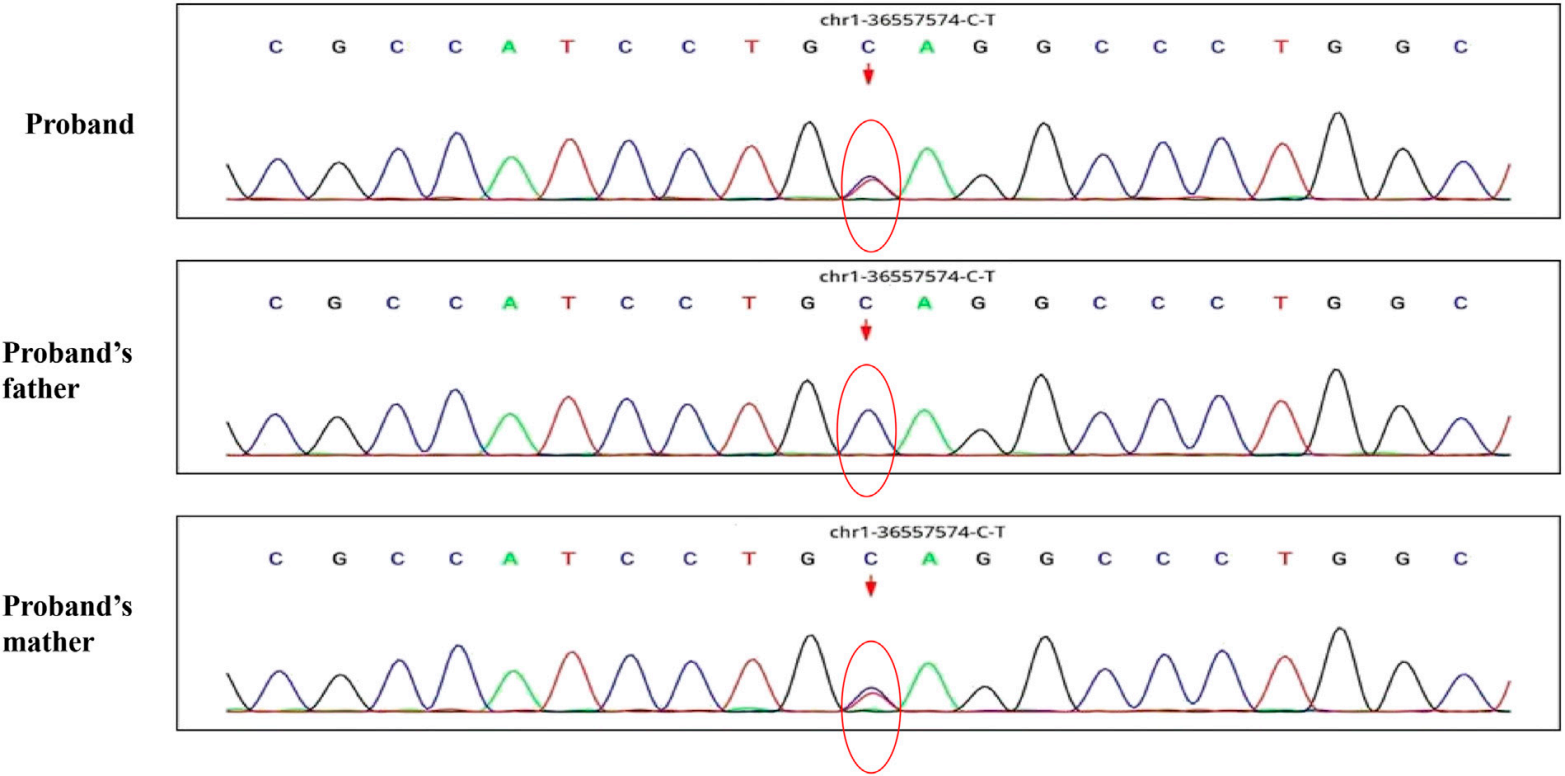
The whole exon sequence of C.580C>T in this child patient and his parents.

**FIGURE 2:**
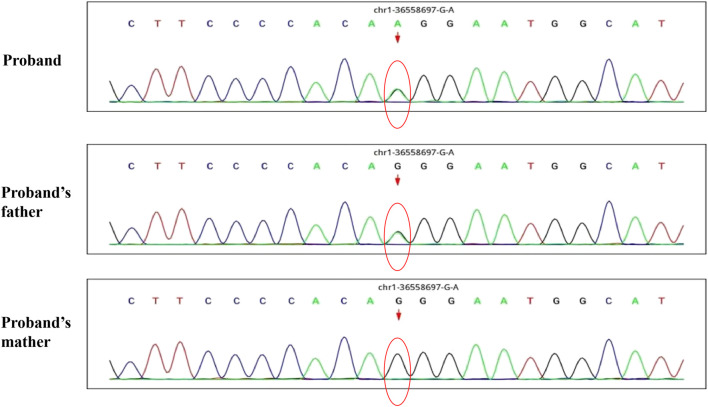
The whole exon sequence of C.803-1G>A in this child patient and his parents.

A 30-month-old male patient was admitted to the Department of Neurology of Wuhan Children’s Hospital in July 2020. His parents were not blood relatives. The infant had developed a respiratory tract infection a week prior, and had had recurrent fever for 4 days. After recovery, he had developed an abnormal broad-based gait that caused him to sway from side to side, especially when walking alone, which became impossible without external support. After admission, a comprehensive examination was conducted. No enlargement of superficial lymph nodes was detected. The shape of the skull was normal and the fontanelle were closed. The eyelids were normal, and the sclera was free of yellow stains. The pupils were equal, rounded, and 3 mm in size, with normal light reflex. A physiological curvature of the spine, but no pathological deformities or reflexes, were noted. Kerning and Brudzinski’s tests were both negative. The patient could not cooperate during the finger-nose test, could not walk in a straight line, and was positive to the Romberg test. Paroxysmal limb jitter had been observed during the sleep period in the prior 2 days, and waggle was present as well. The patient coughed while drinking water. No dyspnea, shortness of breath, cyanosis, developmental delay, or speech impairment were observed. The Gesell Developmental Observation-Revised scale was used to assess developmental level. The results showed that the patient’s developmental level was similar to that of a 15-week-old child. Laboratory biochemical tests were performed. The following parameters were normal: blood RT, serum immunoglobulin M (IgM) for respiratory tract pathogens, serum amyloid protein A, human immunodeficiency virus (HIV) antibodies, syphilis-specific antibodies, liver and kidney functions, electrolytes, and cardiac enzymes. The levels of lactic acid and procalcitonin in the blood were 2.34 mmol/L and 0.08 ng/ml, respectively (reference ranges: 4.5–19.8 mmol/L and <0.05 ng/ml, respectively). Serum thyroid-stimulating hormone levels had decreased to 0.269 μIU/ml (reference range: 0.5–5.0 μIU/ml). After cerebrospinal fluid (CSF) biopsy, the CSF glucose test was positive. The serum and CSF were negative for ganglioside antibodies. The abundance of CSF auto-antibodies fell within the reference range. Lung texture was detected by chest computerized tomography (CT). Electrocardiogram examination showed sinus bradycardia, and QT dispersion was 9 ms. Dysplasia with consistent fluid effusion was observed. Magnetic resonance imaging (MRI) of the cervical, thoracic, and lumbar spine exhibited no obvious abnormalities (Supplementary Figure S1A). No other abnormalities were detected during the first brain MRI after admission. Electroencephalogram (EEG) examination showed abnormal EEG results. During the waking period, the posterior lobe exhibited slow-wave activity. During the sleep period, multifocal spikes and spike-wave activity were observed, and no abnormal discharge in the parental identification event existed (Supplementary Figure S1B). The results of electromyography (EMG) showed that motor sensory nerve conduction was normal. Based on these test results, the child was diagnosed with acute cerebellar ataxia. Gamma globulin (2 g/kg) was administered as a shock therapy, but the symptoms did not improve, and worsened after low-dose hormone therapy (20 mg/kg per day). Brain MRI was performed again, and showed slightly wider sulci on the cerebellar surface (Supplementary Figure S2). The patient died due to convulsions several months later. After consultation with the parents, we found that the patient’s biological father, grandfather, and uncle all had a history of convulsions. Since the patient had been stunted since birth, we speculated that mutations in metabolism-related factors contributed to the disease. Therefore, whole exon sequencing was conducted on the patient and his biological parents to identify the mutation responsible for the observed phenotype, and RNA sequencing was performed to examine the functions of the mutations. The results indicated that the child carried two novel compound heterozygous variations in the *ADPRS* gene: c.580C>T (Figure 1) and c.803-1G>A (Figure 2) inherited from his parents (Table 1). The father was wild type and heterozygous for the c.580C>T and c.803-1G>A mutations, respectively. On the other hand, the mother was a carrier of the c.580C>T variant, but had wildtype genotype for the c.803-1G>A mutation. The c.580C>T mutation (p.Gln194Ter; chr1:36557574; NM_017825. 3:c.508C>T; hybrid) is a frameshift deletion variant in exons 4/6 that was first reported in 2018. The cytosine at position 580 is substituted by a thymidine; because of this, the codon for glutamine (Gln) at position 194 is changed into a premature termination codon, resulting in a shorter protein (Supplementary Figure S3). The allele frequency of this variant is not reported in the Genome Aggregation Database (gnomAD; https://gnomad.broadinstitute.org). This variant is also not present in the ClinVar (https://www.ncbi.nlm.nih.gov/clinvar/) and the Human Gene Mutation Database (HGMD; http://www.hgmd.cf.ac.uk/ac/index.php). The analysis of the trans position of the c.580C>T variant indicated that the frequency of the cytosine-containing sequence is 92%, while that of the thymidine-containing variant is 8%. This suggests that most of the transcribed thymidine-containing RNA may be degraded through nonsense-mediated mRNA decay (Supplementary Figure S4). Because the c.580 C>T variant affects the coding region of *ADPRS*, we evaluated the pathogenicity of the variant through the Combined Annotation Dependent Depletion scoring tool (https://cadd.gs.washington.edu) and MutationTaster (http://www.mutationtaster.org/). The results of these analyses are presented in Tables 2, 3. In addition, the ConSurf Server (https://consurf.tau.ac.il) was used to assess residue conservation. This analysis suggested that Gln at position 194 was highly conserved. The UCSC Genome Browser (https://genome.ucsc.edu) indicated that the cytosine at position 580 is highly conserved in primates (Supplementary Figure S5). The 3D protein structures were visualized with the SWISS-MODEL Server (https://swissmodel.expasy.org), and the structures of the wildtype and mutated protein are visualized in Supplementary Figures S6A,B. According to the guidelines of the American College of Medical Genetics and Genomics (ACMG), the c.580 C>T mutation was classified as pathogenic (PVS1 + PM2_Supporting + PS3). In the c.803-1G>A mutation the guanine at position 803–1 changes into an adenosine (Supplementary Figure S7). To date, the minor allele frequency of this variant in gnomAD is 0. This mutation is also not reported in the ClinVar and HGMD databases. RNA sequencing revealed that this variant is a splice-site mutation that leads to the retention of intron 5 in the final mRNA product. Furthermore, this variant destroys the shear structure “GT-AG,” resulting in the failure to identify the correct shear position (Supplementary Figure S8). According to the ACMG guidelines, c.803-1G>A was classified as pathogenic (PS3 + PM2_Supporting + PM3). In addition, both mutations were categorized as likely pathogenic (Hanai et al., 2004).

**TABLE 1:** The results of whole exon sequence of c.580C>T and c.803-1G>A.

Gene symbol	Chromosome location	Variant	Proband	Father	Mother	Gene frequency	Source
*ADPRS*	chr1: 36557574	NM_017825. 3:c.580C>T (p. Gln194Ter), Exon4|6	Heterozygosis	Wide type	Heterozygosis	NA	Mother
*ADPRS*	chr1: 36558697	NM_017825. 3:c.803-1G>A, Intron5|5	Heterozygosis	Heterozygosis	Wide type	0	Father

**TABLE 2:** The predictive result of CADD score.

Variant	c.580C>T	c.803-1G>A
Transcript	ENST00000373178	ENST00000373178
Gene symbol	ADPRS	ADPRS
Prediction	Deleterious (fs/PTC)	Deleterious
Prediction problem Splice site change	No	None
Amino acid changes	Q194Ter	No
Variant type	Single base exchange	None
Protein length	NMD	N/A
Features at a glance	Amino acid sequence changed	Splice site lost
	NMD	
	Protein features (might be) affected	

**TABLE 3:** The predictive result of MutationTaster.

Chromosome	1	1
Position	36557574	36558697
Reference	C	G
Alteration	T	A
Score	41	35

## Discussion

To the best of our knowledge, this is the first report of CONDSIAS caused by two compound heterozygous *ADPRS* variants: c.580C>T (p.Gln194Ter) and c.803-1G>A. Our report suggests that PAR removal is particularly important in the etiology of CONDSIAS. In humans, two PAR-degrading enzymes exist: ARH3 and poly(ADP-ribose) glycohydrolase (PARG; OMIM: 603501). PARG and ARH3 hydrolyze the glycosidic bond between PAR polymers. ARH3 has the ability to cleave the bond between serine and ADP-ribose, but PARG does not. Both enzymes prevent the accumulation of PAR (Andrabi et al., 2006; Mascalchi et al., 2018). Mashimo et al. discovered that PARG and ARH3 act synergistically to regulate nuclear and cytoplasmic degradation of PAR after H_2_O_2_ exposure, thereby preventing the release and accumulation of apoptosis-inducing factor (Mashimo et al., 2013). Furthermore, Mashimo et al. also reported the case of a family with an *ADPRS* mutation that resulted in a truncated, inactive protein. In this case, an 8-year-old child presented with progressive neurodegeneration, while an older sibling exhibited a mild behavioral phenotype. Parthanatos was also observed in the neurons of the patient’s deceased sibling. In addition, the child’s fibroblasts, as well as ARH3-deficient mice, were shown to be more sensitive to PAR accumulation and cell death induced by H_2_O_2_ stress and cerebral ischemia/reperfusion, respectively (Mashimo et al., 2019). Ghosh et al. also reported six independent families with *ADPRS* variants. Whole exon sequencing revealed different homozygous mutations, including c.530C>T in exon 4, a 5-bp deletion and c.316C>T in exon 3, c.1000C>T in exon 6, c.235A>C in exon 2, and c.100G>A in exon 1. All patients showed a common CONDSIAS phenotype. Most of the examined patients were in their early childhood and in the acute onset of infection-related stress. Two-thirds of the patients presented with epilepsy, which progressed gradually. All patients died in adolescence. A few children with late onset (10–13 years old) exhibited neurological degeneration without epilepsy, and only one patient survived to the age of 30 (Ghosh et al., 2018). Epilepsy is common in early-onset patients and may be a risk factor for poor prognosis and death (Hanai et al., 2004). Danhauser et al. also reported clinical symptoms such as developmental delay, abnormal gait, seizures, ataxia, and other neurodegenerative lesions. Additionally, patients could also present with hearing loss, microencephaly, respiratory function incompetence, facial muscle clonus, and oculomotor abnormalities (Danhauser et al., 2018). Lastly, Xu et al. reported the case of a female child with epilepsy-ataxia syndrome caused by stress and worsened by *ADPRS* variation. A shear-sensitive c.301G>T homozygous mutation in *ADPRS* was found in both the patient and her sister, and was inherited from their parents. Paroxysmal gait abnormalities, limb weakness, ataxia, peripheral neuropathy, and cerebellar atrophy were observed after an infection at the age of 2, and lead to death by respiratory failure at the age of 4. Similarly, the patient’s sister presented with gait abnormalities and developmental delay from 18 months of age. After an infection at the age of 13, she developed epilepsy and disorders of consciousness, and died after treatment failure (Xu et al., 2020). The symptoms described for our patient are in the line with those described by these previous reports. In conclusion, the c.580C>T mutation detected in our patient leads to the deletion of all amino acids after position 194. As a consequence, intact protein structure and enzyme activity may be lost, which could have led to the development of ataxia, epilepsy, developmental delay, gait instability, and the other symptoms observed in our patient.

## Data Availability

The datasets for this article are not publicly available due to concerns regarding participant/patient anonymity. Requests to access the datasets should be directed to the corresponding authors.

## Supplementary Material

Supplementary Figure S1 Patient brain MRI **(A)** and EEG **(B)** performed at admission.

Supplementary Figure S2 Brain MRI after therapy of this child patient.

Supplementary Figure S3 The predictive regulation mode pattern of C. 580C>T.

Supplementary Figure S4 The genetic analysis result of C. 580C>T by RNA-Seq **(A–C)**.

Supplementary Figure S5 The position of 194 Gln in amino acid sequence **(A)**; The evolutionary conservation of C nucleotide in primates **(B)**; The evolutionary conservation of 194 Gln **(C)**.

Supplementary Figure S6 The 3D model of wide type **(A)** and mutation type **(B)** of protein.

Supplementary Figure S7 The predictive regulation mode pattern of C.803-1G >A.

Supplementary Figure S8 The genetic analysis result of C.803-1G >A by RNA-Seq **(A–D)**.
