# Supplementary figures and images for: Child-Onset Cerebellar Ataxia Caused by Two Compound Heterozygous Variants in ADPRS Gene: A Case Report
Source: Front Genet. 2022 May 19;12:788702. doi: 10.3389/fgene.2021.788702 (PMC9160522; doi:10.3389/fgene.2021.788702)

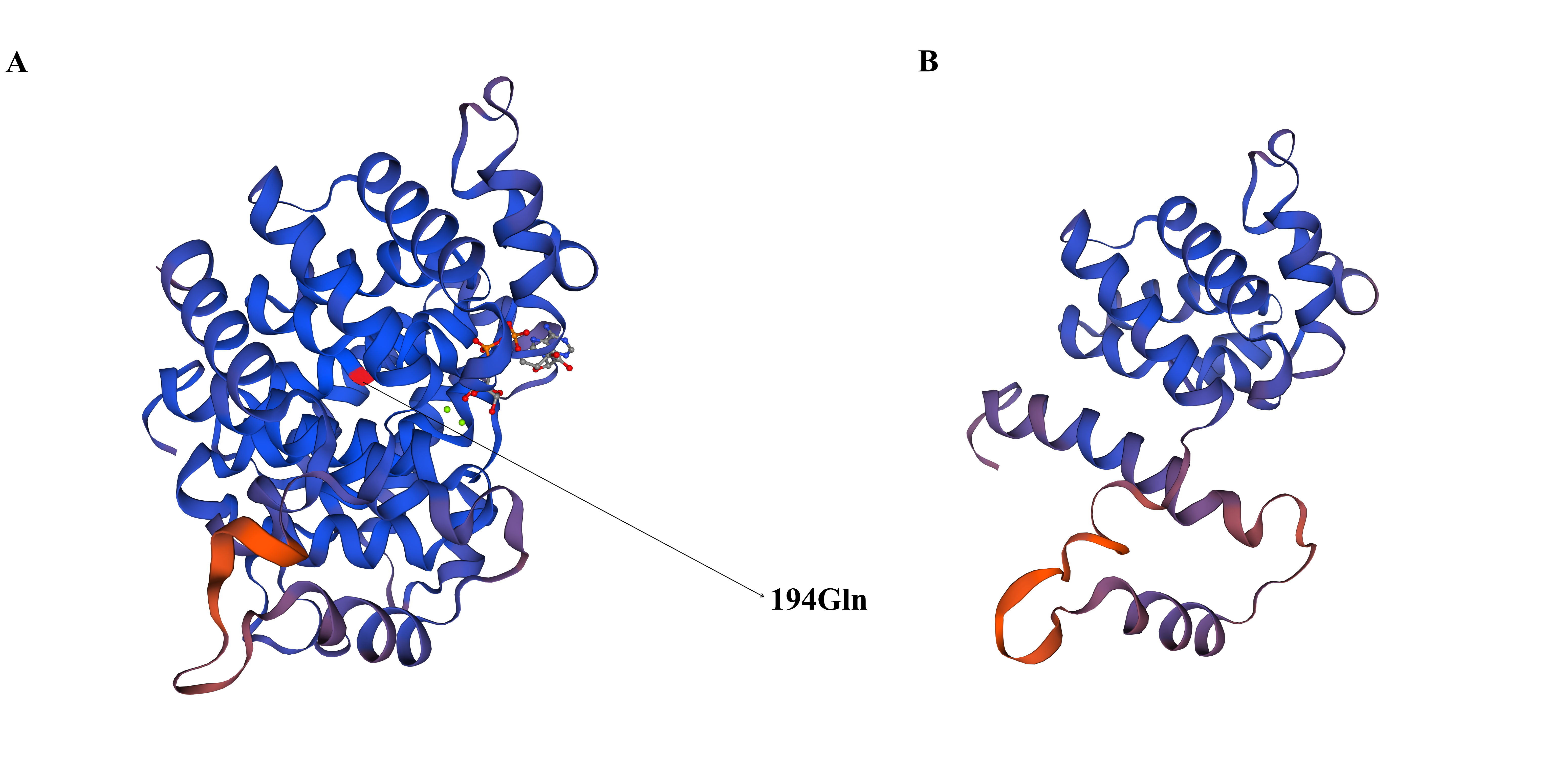

Supplement: Supplementary file 1 [file Image6.TIF]

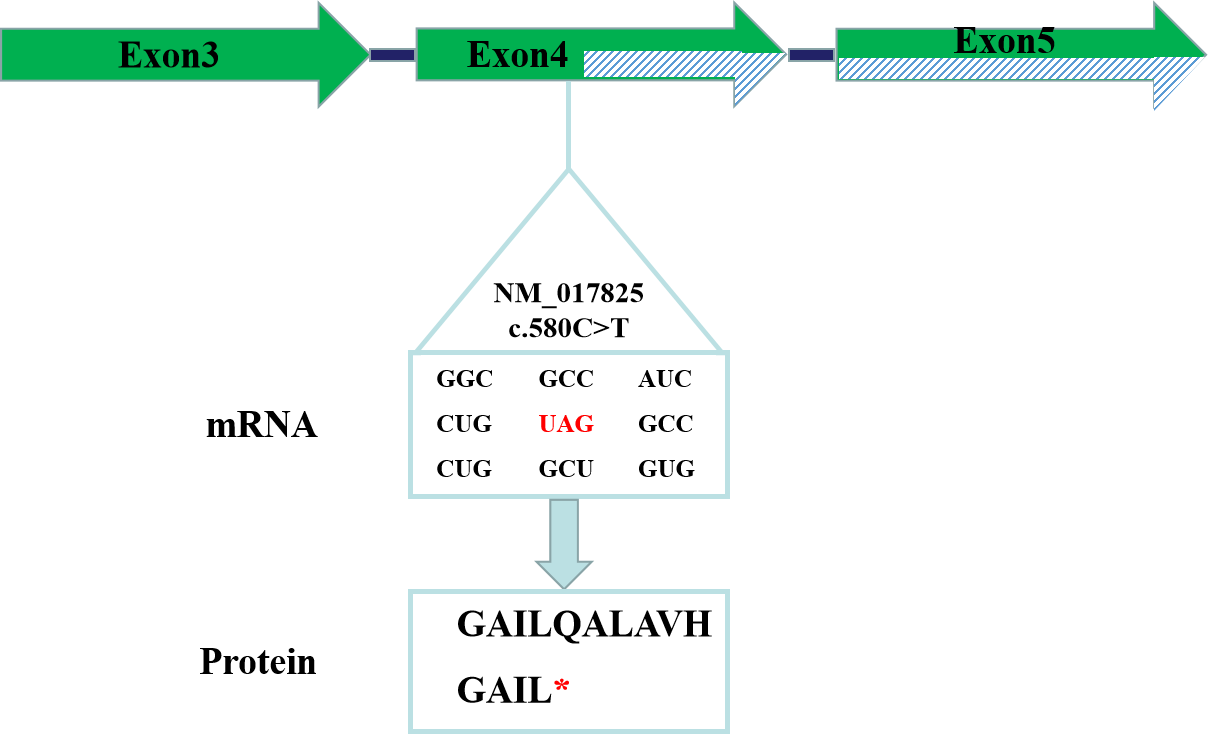

Supplement: Supplementary file 2 [file Image3.TIF]

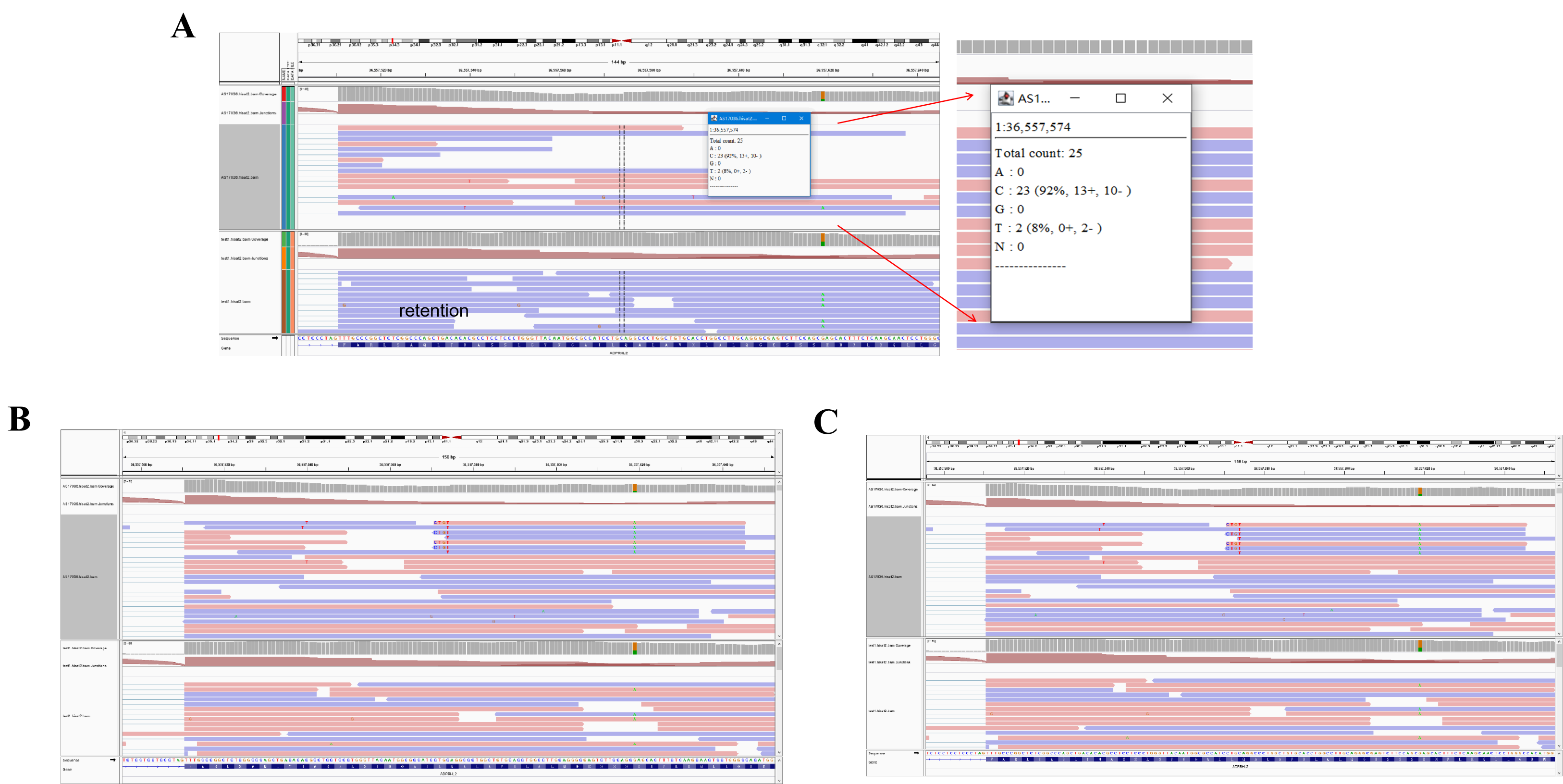

Supplement: Supplementary file 3 [file Image4.TIF]

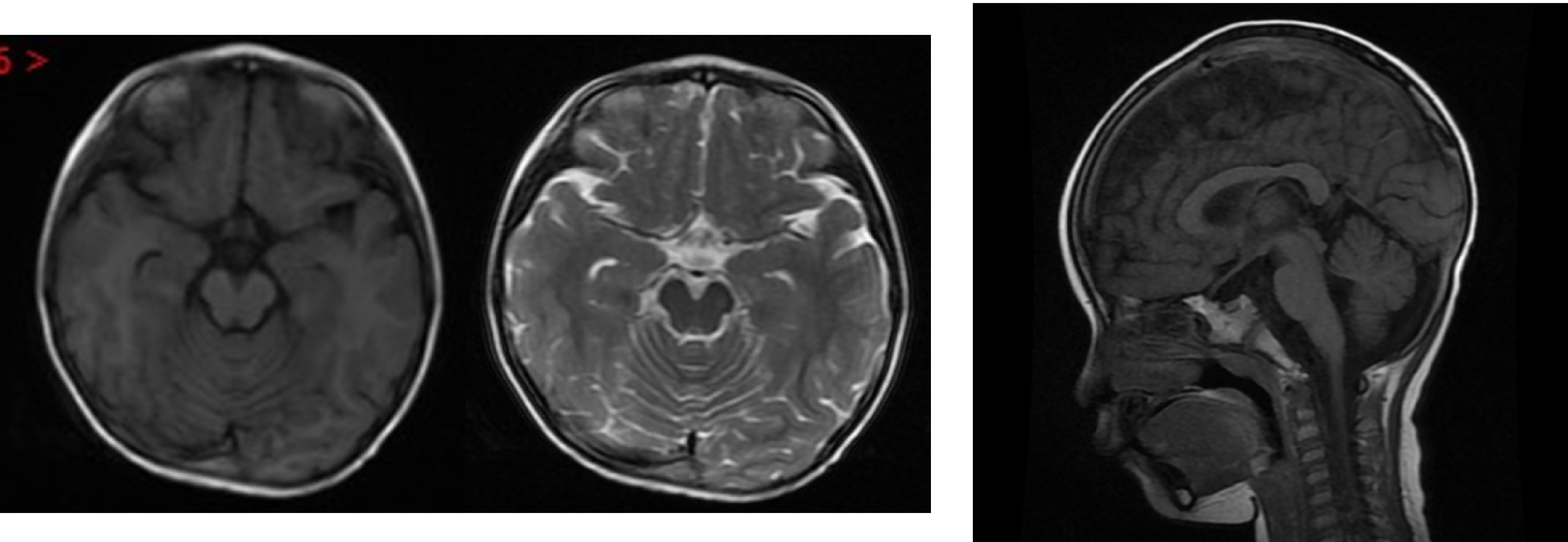

Supplement: Supplementary file 4 [file Image2.TIF]

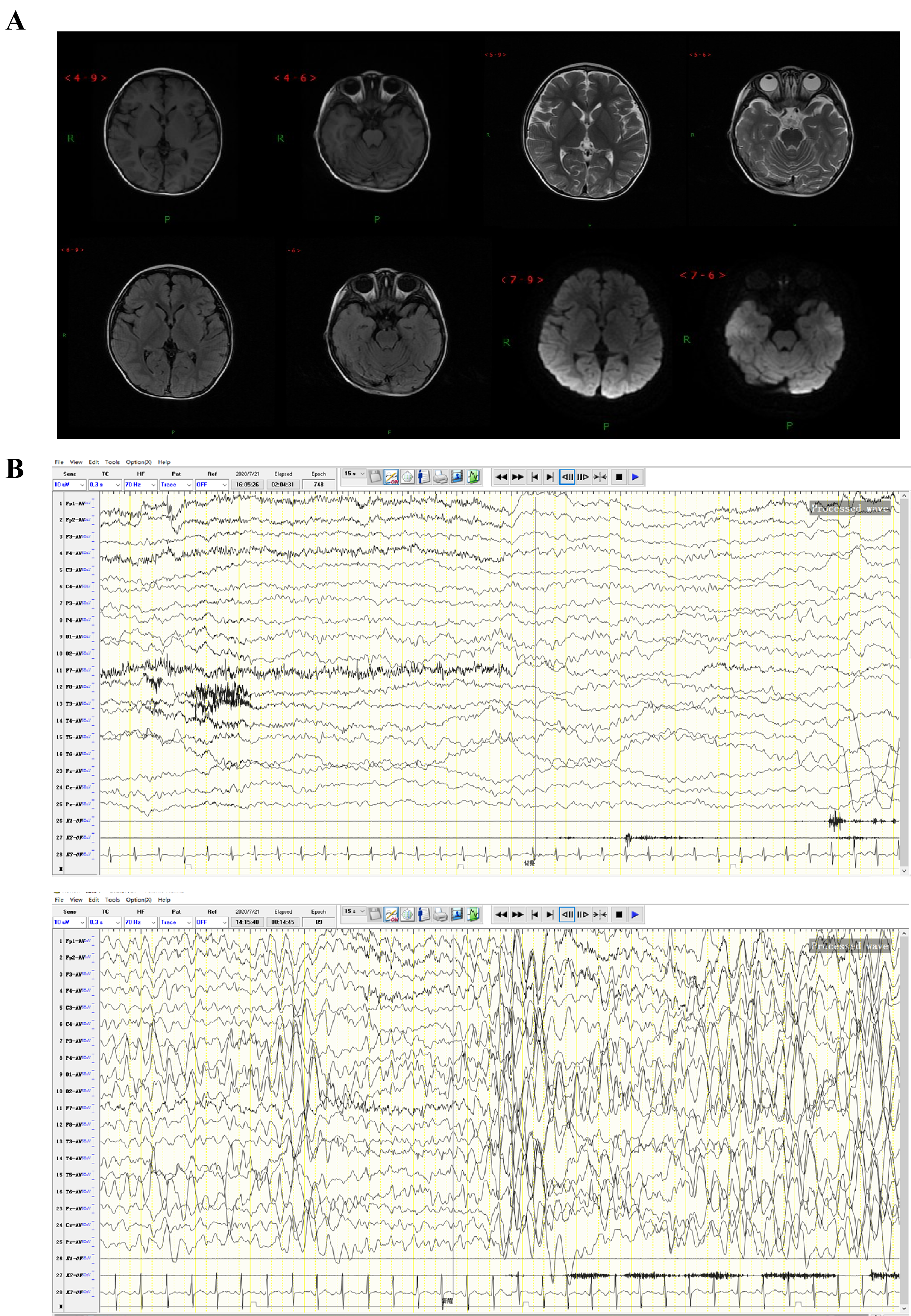

Supplement: Supplementary file 5 [file Image1.TIF]

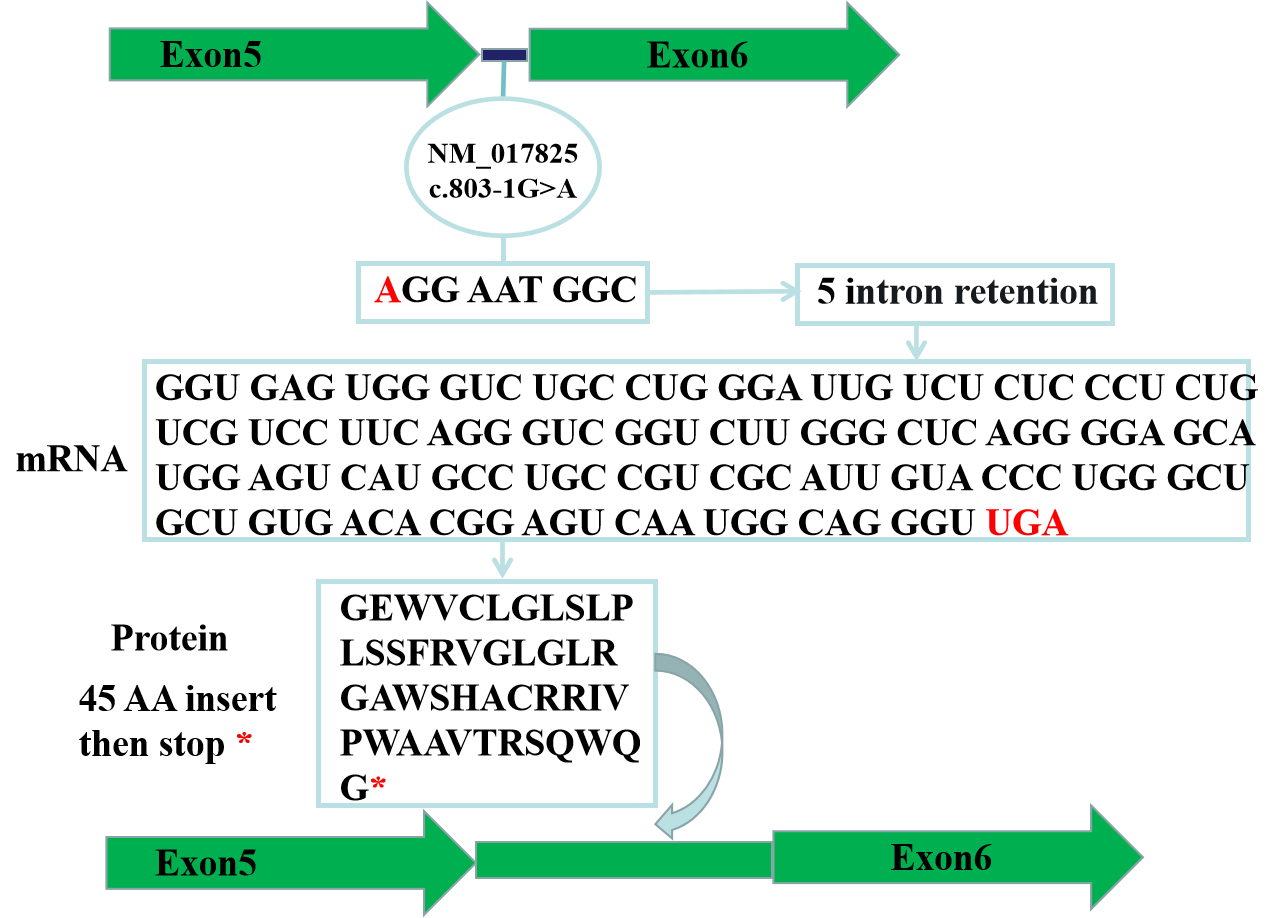

Supplement: Supplementary file 6 [file Image7.TIF]

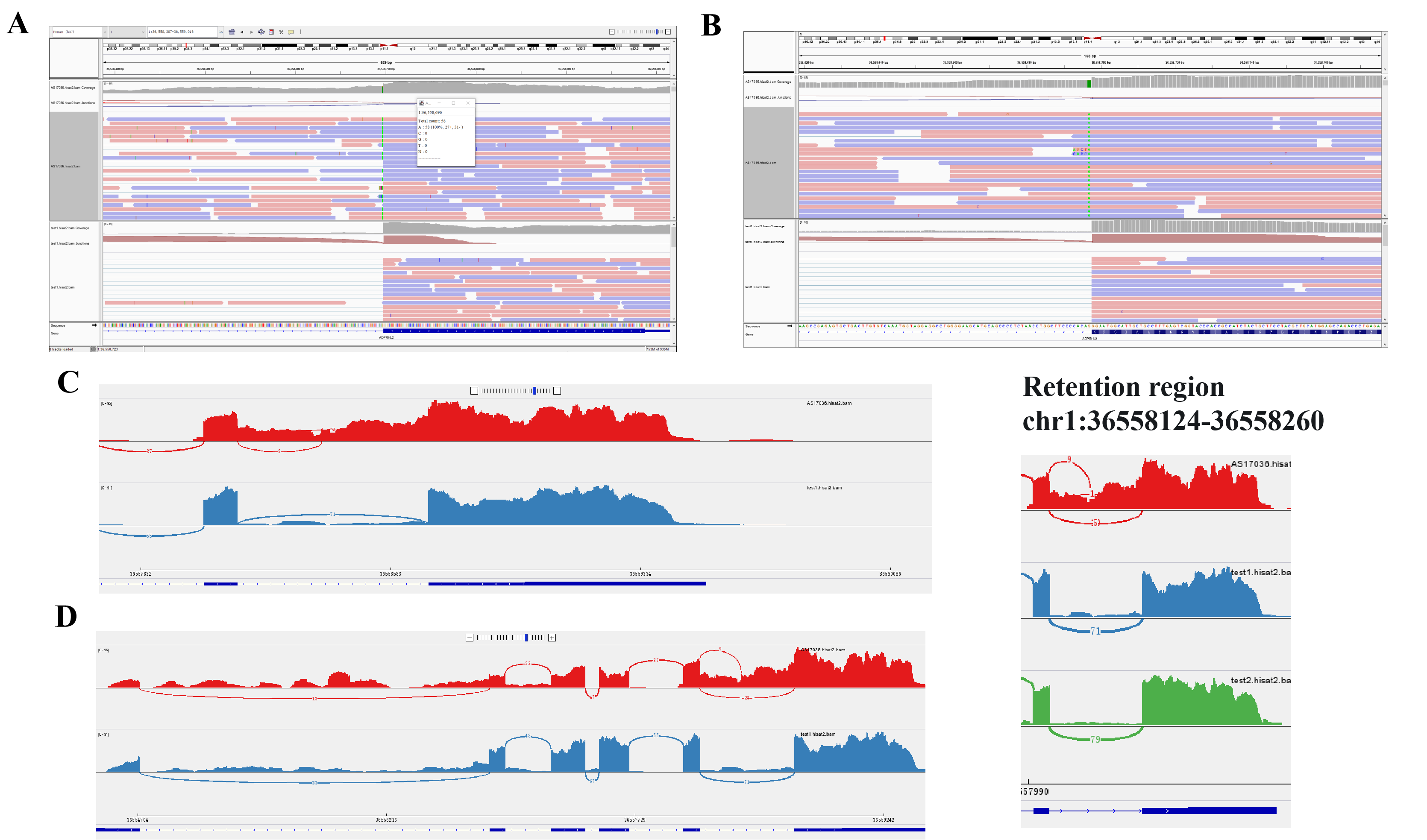

Supplement: Supplementary file 7 [file Image8.TIF]

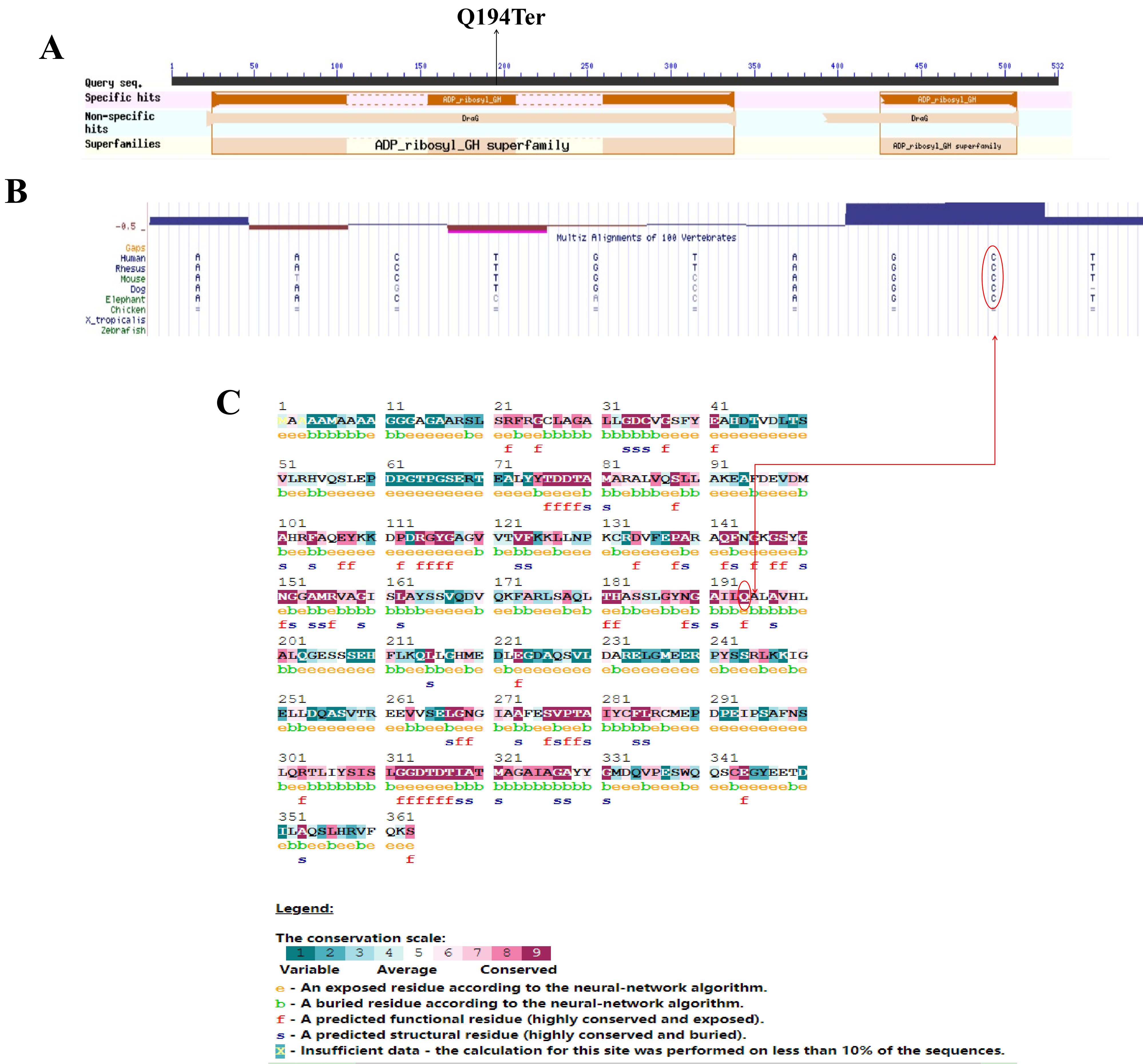

Supplement: Supplementary file 8 [file Image5.TIF]
